# Structural studies and functional engineering of NanX: an anhydro‐sialic acid transporter from *Escherichia coli*

**DOI:** 10.1002/2211-5463.70310

**Published:** 2026-07-14

**Authors:** Michael C. Newton‐Vesty, Kelsi R. Hall, Zachary D. Tillett, Liam S. Turk, James S. Davies, Michael J. Currie, Hamish G. Brown, Sepideh Valimehr, Peter D. Mace, Andrew E. Whitten, Santosh Panjikar, Eric Hanssen, Rachel A. North, Renwick C.J. Dobson

**Affiliations:** ^1^ Biomolecular Interaction Centre, School of Biological Sciences, MacDiarmid Institute for Advanced Materials and Nanotechnology University of Canterbury Christchurch New Zealand; ^2^ ARC Centre for Cryo‐Electron Microscopy of Membrane Proteins, Bio21 Molecular Science and Biotechnology Institute, Department of Biochemistry and Molecular Biology University of Melbourne Parkville Australia; ^3^ Computational and Structural Biology Division Victor Chang Cardiac Research Institute Sydney Australia; ^4^ Ian Holmes Imaging Centre, Bio21 Molecular Science and Biotechnology Institute The University of Melbourne Australia; ^5^ ARC Industrial Transformation Training Centre for Cryo‐Electron Microscopy of Membrane Proteins University of Melbourne Parkville Australia; ^6^ Biochemistry Department, School of Biomedical Sciences University of Otago Dunedin New Zealand; ^7^ Australian Centre for Neutron Scattering (ACNS) Australian Nuclear Science and Technology Organisation (ANSTO) Lucas Heights Australia; ^8^ Australian Synchrotron, ANSTO Clayton Australia; ^9^ Department of Biochemistry and Molecular Biology Monash University Melbourne Australia; ^10^ Department of Biochemistry & Pharmacology, Bio21 Molecular Science and Biotechnology Institute The University of Melbourne Australia; ^11^ School of Medical Sciences, Faculty of Medicine and Health University of Sydney Australia

**Keywords:** *Escherichia coli*, major facilitator superfamily (MFS) transporters, NanT, NanX, sialic acid

## Abstract

NanT and NanX are bacterial transporters that import the sialic acids, *N*‐acetylneuraminate and 2,7‐anhydro‐*n*‐acetylneuraminate, respectively. Here, we used complementary biophysical and computational approaches to structurally characterise *Escherichia coli* NanX. Size exclusion chromatography, analytical ultracentrifugation and low‐resolution cryo‐electron microscopy reveal that NanX exists in both monomeric and dimeric states following purification. Molecular modelling and substrate docking identify key residues likely involved in 2,7‐anhydro‐*n*‐acetylneuraminate recognition. Using this information, we engineered a mutant NanX transporter that can import the NanT‐specific substrate, *N*‐acetylneuraminate, which we verified using a bacterial growth assay. These data identify amino acids involved in major facilitator superfamily mediated sialic acid transport and offer a new research perspective of its metabolism.

## Graphical abstract

Biophysical characterisation shows that NanX, a membrane transport protein from the major facilitator superfamily (MFS), forms both monomers and dimers after purification. alphafold modelling and substrate docking provide information on residues likely involved in substrate recognition for NanX and another MFS member, NanT. Mutagenesis yields a NanX variant that transports a NanT‐specific substrate and is confirmed by bacterial growth assays.

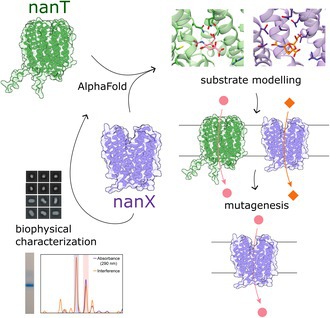

**Abbreviations**
2,7‐anhydro‐Neu5Ac: 2,7‐anhydro‐*n*‐acetylneuraminate
ABC: ATP‐binding cassette transporter
CMC: critical micelle concentration
DDM: n‐Dodecyl‐β‐*d*‐maltopyranoside
*Ec*NanT: the Neu5Ac transporter from *Escherichia coli*
*Ec*NanX: The 2,7‐anhydro‐Neu5Ac transporter from *Escherichia coli*
*f/f*_*0*_
: frictional ratio
GFP: green fluorescent protein
H^+^
: proton
LMNG: Lauryl maltose neopentyl glycol
MFS: major facilitator superfamily
Na^+^
: sodium ion
Neu5Ac: *N*‐acetylneuraminate
Neu5Ac2en: 2‐Deoxy‐2,3‐dehydro‐*n*‐acetylneuraminate
RFU: relative fluorescence units
S: sedimentation coefficient
SDS/PAGE: sodium dodecyl/sulfate polyacrylamide gel electrophoresis
SEC: size‐exclusion chromatography
SSS: sodium solute symporter
TRAP: tripartite ATP‐independent periplasmic

Sialic acids are a family of over 50 structurally related α‐keto acid sugars that are derived from a nine‐carbon neuraminate backbone [1, 2]. Sialic acids are ubiquitous in vertebrates and occupy terminal positions of cellular glycoconjugates on mucosal surfaces [3]. These carbohydrates play crucial roles in cellular recognition, adhesion and signalling [3]. Many pathogenic bacteria use host‐displayed sialic acids to gain a competitive advantage, and some have evolved mechanisms to incorporate host sialic acids into their own glycoconjugates to evade the host immune system through molecular mimicry [4, 5, 6]. Most sialic acid‐metabolising bacteria are host‐dependent for sialic acid; only a few are capable of *de novo* synthesis [7].

Bacterial catabolism of sialic acid begins with its sialidase‐mediated cleavage from host glycoconjugates [8, 9, 10]. Many sialic acid‐utilising species do not express sialidases and instead rely on those produced by the host or other bacteria [8, 11, 12]. Sialidases typically cleave sialic acid into three forms (Fig. 1A): *N*‐acetylneuraminate (Neu5Ac), 2,7‐anhydro‐*n*‐acetylneuraminate (2,7‐anhydro‐Neu5Ac) and 2‐deoxy‐2,3‐dehydro‐*n*‐acetylneuraminate (Neu5Ac2en) [13, 14, 15, 16]. These access the periplasm of Gram‐negative bacteria through general outer membrane porins or sialic acid‐specific porins that include NanC and the NanOU system [17, 18, 19, 20]. Transport across the bacterial plasma membrane is mediated by transporters from four superfamilies (Fig. 1B). These include the ATP‐binding cassette (ABC) [21, 22, 23, 24, 25], tripartite ATP‐independent periplasmic (TRAP) [26, 27, 28, 29, 30, 31, 32, 33, 34] sodium solute symporter (SSS) [35, 36, 37] and major facilitator superfamily (MFS) transporters [8, 38] (reviewed in [11, 39, 40, 41, 42]). Sialic acid MFS transporters remain structurally uncharacterised, and the transport of anhydro‐sialic acid derivatives—2,7‐anhydro‐Neu5Ac and Neu5Ac2en—is contested, with studies offering different accounts [43, 44].

**Fig. 1:**
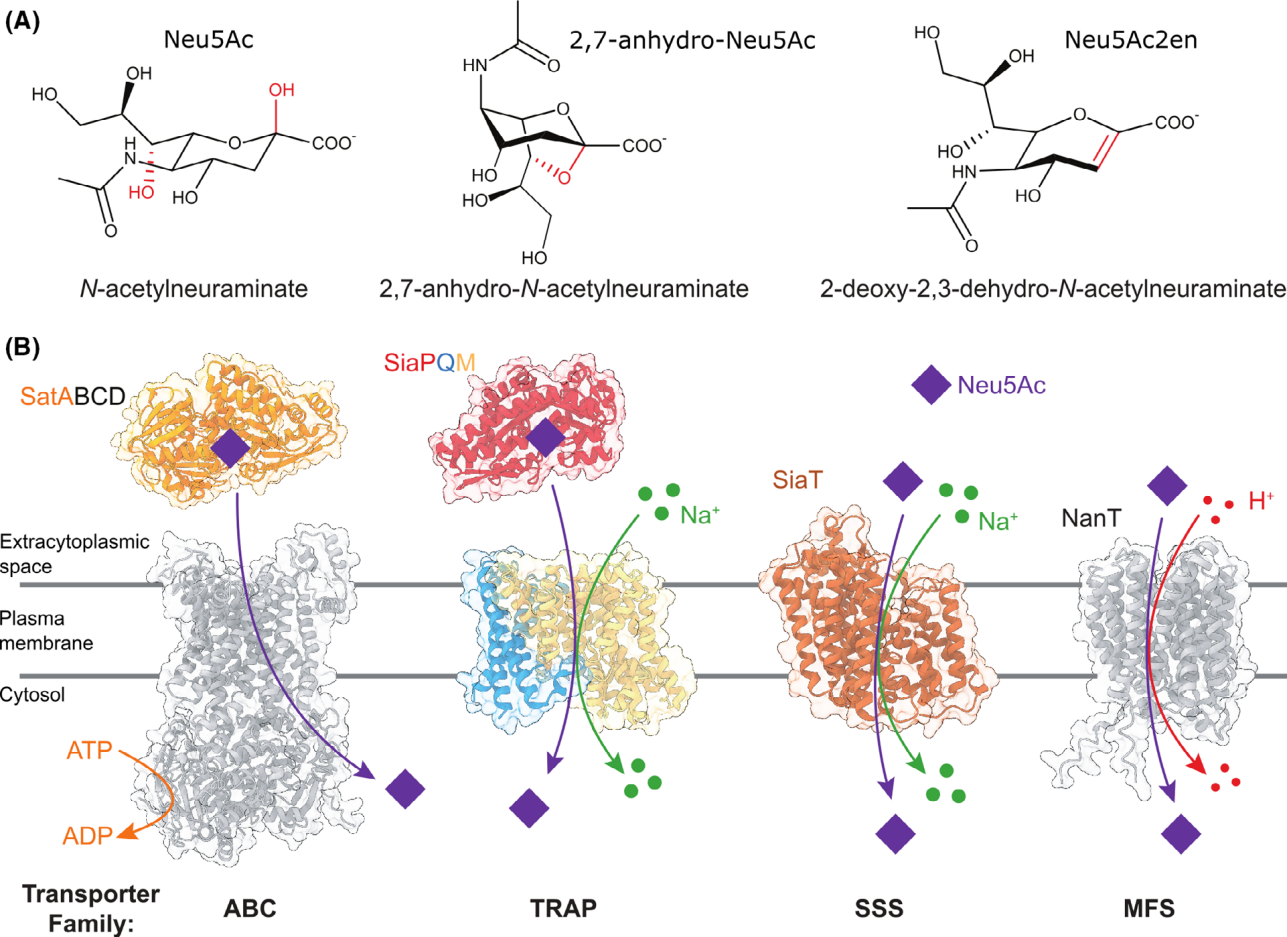
Structures of sialic acids and sialic acid transporters from four transporter families. (A) Chemical structures of *N*‐acetylneuraminate (Neu5Ac, purple diamond), 2,7‐anhydro‐*n*‐acetylneuraminate (2,7‐anhydro‐Neu5Ac), and 2‐deoxy‐2,3‐dehydro‐*n*‐acetylneuraminate (Neu5Ac2en) are shown. Structural differences are coloured in red. (B) Four families of sialic acid transporters and representative structures (PDB IDs: *Haemophilus ducreyi* SatA, 5Z99, *Photobacterium profundum* SiaP, 7T3E, and SiaQM, 7QHA, and *Proteus mirabilis* SiaT, 5NVA). Transporters coloured in grey have not been structurally characterised, and alphafold2 models [59] are shown instead (UniProt IDs: *H. ducreyi* SatBCD, Q7VL15/Q7VL16/Q7VL17, *Escherichia coli* NanT, P41036). Models are visualised using chimerax [77].

The ability to import multiple forms of sialic acid is proposed to provide bacteria with a competitive advantage in nutrient‐limited environments [41, 42, 45, 46]. *Escherichia coli* possess the 2,7‐anhydro‐Neu5Ac transporter, NanX (*Ec*NanX), and the Neu5Ac transporter, NanT (*Ec*NanT) [38, 44, 46, 47]. Similarities between *Ec*NanX and *Ec*NanT are evident; emerging from the same evolutionary background, both are MFS transporters and both couple substrate translocation with energy obtained from a H^+^ gradient [42, 44, 48]. *Ec*NanX and *Ec*NanT are predicted to comprise two sets of transmembrane helix bundles for a total of 12 transmembrane helices in *Ec*NanX [42] and 14 in *Ec*NanT [38] and share 55% sequence similarity (35% identity) [46].

Here, we focus on a structural characterisation of the 2,7‐anhydro‐Neu5Ac transporter from *E. coli* (*Ec*NanX). Biophysical approaches demonstrate that purified recombinant *Ec*NanX forms both monomeric and dimeric forms. Using *in silico* methods, we analyse its substrate‐binding site and compare it to that of *Ec*NanT. We identify several differences in the substrate‐binding pocket and alter the substrate specificity of *Ec*NanX to be able to transport the *Ec*NanT substrate, Neu5Ac. Bacterial catabolism of sialic acid by pathogenic bacteria has been shown to play a role in colonisation of specific niches, such as the gut [21, 49, 50, 51]. Further understanding the mechanism of sialic acid import through the work of this study and studies that follow may inform methods to develop antimicrobials targeting this pathway.

## Materials and methods

### Cloning and mutagenesis

DNA constructs were synthesised by GenScript. For *Ec*NanX‐GFP, the gene encoding *E. coli* NanX (*Ec*NanX) was cloned into a pWarf(−) [52] expression vector in‐frame with a C‐terminal human rhinovirus 3C (HRV 3C) protease cleavage site, green fluorescent protein (GFP) and octa‐histidine tag (His‐tag). For *Ec*NanX‐His, the gene encoding *Ec*NanX, as well as an HRV 3C protease cleavage site and a His‐tag were cloned into a pET28a(+) vector.

For bacterial growth experiments, the gene encoding *E. coli* NanT (*Ec*NanT) was cloned into a pJ422 vector to form the pJ422_*EcnanT* construct. NEBuilder HiFi DNA Assembly (New England Biolabs) was used to clone *Ec*NanX into a pJ422 vector to make the pJ422_*EcnanX* construct. Site‐directed mutagenesis [53] was employed to generate the pJ422_*EcnanX* mutant constructs. The sequences of primers used are presented in Table S1.

### Protein expression and purification

For expression of all *Ec*NanX constructs, plasmids were transformed into *E. coli* Lemo21 (DE3) cells (New England Biolabs, Ipswich, MA, USA). Media were supplemented with kanamycin (50 μg·mL^−1^) and chloramphenicol (34 μg·mL^−1^) throughout. A single colony was used to inoculate Terrific Broth (TB) medium and grown overnight at 37 °C, shaking at 180 rpm.

For large‐scale expression of the *Ec*NanX constructs, TB medium was inoculated with the overnight culture and cells were grown at 37 °C, shaking at 180 rpm to an OD_600_ of 1.6–1.8. Protein expression was induced with 1 mm isopropyl β‐*d*‐1‐thiogalactopyranoside (IPTG) for 18 h at 26 °C, shaking at 180 rpm (*Ec*NanX‐GFP) or 3 h at 37 °C, shaking at 180 rpm (*Ec*NanX‐His). Cells were collected by centrifugation at 8000 ***g*** for 5 min at 4 °C and stored at −80 °C before membrane preparation. Cell pellets were homogenised in PBS pH 7.4, 0.5 mg·mL^−1^ lysozyme and 0.2 mm phenylmethylsulfonyl fluoride (PMSF) at a 3 : 1 (mL : g) ratio. The resuspended cells were lysed by ultrasonication at 70% amplitude in 0.5 s on, 0.5 s off cycles for 30 min on ice, using a Hielscher UP200S Ultrasonic Processor. Cell debris was removed by two centrifugation steps at 22 000 ***g*** and 4 °C for 25 min. Membranes were harvested by ultracentrifugation at 250 000 ***g*** in a 50.2 Ti rotor (Beckman Coulter, Brea, CA, USA) at 4 °C for 3 h.

For fluorescence size‐exclusion chromatography (FSEC), membranes containing *Ec*NanX‐GFP were resuspended to 5% (w/v) in PBS pH 7.4 and aliquoted. To each aliquot, n‐dodecyl‐β‐*d*‐maltoside (DDM, Anatrace, Maumee, OH, USA) or lauryl maltose neopentyl glycol (LMNG, Anatrace, Maumee, OH, USA) was added to a final concentration of 2% (w/v). Membranes were solubilised with gentle agitation at 4 °C for 2 h before insoluble material was removed by ultracentrifugation at 160 000 ***g*** in a S100AT3 rotor (Beckman Coulter) at 4 °C for 45 min. To assess monodispersity, 240 μL of the soluble fraction of each sample was injected onto a Superose 6 Increase 10/300 GL size‐exclusion column (Cytiva, Wilmington, DE, USA) equilibrated in the relevant buffer at 4 °C. The fluorescence of GFP was monitored using a Dionex UltiMate 3000 FLD‐3100 fluorescence detector (Thermo Fisher Scientific, Waltham, MA, USA) with excitation and emission wavelengths of 488 nm and 512 nm, respectively. Samples were incubated at 4 °C for 48 h before a further 240 μL was injected to assess monodispersity over time. Elution profiles were normalised to the highest value before being shown in the corresponding figures.

*Ec*NanX‐His membranes were solubilised in PBS pH 7.4, 6% (v/v) glycerol, 5 mm β‐mercaptoethanol, 1 mm PMSF and 2% (w/v) LMNG at 4 °C for 2 h. Insoluble material was removed by ultracentrifugation at 160 000 ***g*** in a 50.2 Ti rotor at 4 °C for 1 h.

*Ec*NanX‐His was purified by immobilised metal affinity chromatography (IMAC) using a 5 mL HisTrap HP column (Cytiva) equilibrated with equilibration buffer (PBS pH 7.4, 6 % v/v glycerol, 5 mm β‐mercaptoethanol, 20 mm imidazole and 0.01% w/v LMNG at 4 °C). Solubilised material was loaded onto the column and washed with at least 20 column volumes of equilibration buffer before bound protein was eluted with equilibration buffer supplemented with 500 mm imidazole.

Fractions containing *Ec*NanX were pooled and concentrated for size‐exclusion chromatography (SEC) using 50‐kDa molecular weight cut‐off concentrators (Millipore, Merck KGaA, Darmstadt, Germany). SEC was conducted using a HiLoad 16/60 Superdex 200 size‐exclusion column (Cytiva) in buffer comprising 50 mm Tris–HCl pH 8.0, 150 mm NaCl and 0.002% (w/v) LMNG at 4 °C. Elution fractions containing *Ec*NanX were pooled, concentrated and snap‐frozen in liquid nitrogen for storage at −80 °C if not being used immediately.

### Single particle cryo‐electron microscopy

Purified *Ec*NanX was incubated with amphipol A8‐35 (Anatrace, Maumee, OH, USA) at a 1 : 5 (w/w) ratio for 2 h before the addition of 100 mg·mL^−1^ Bio‐Beads SM‐2 resin (Bio‐Rad, Hercules, CA, USA). The mixture was then incubated overnight at 4 °C with gentle agitation to remove detergent. After exchange, SEC was performed using a 24 mL Superdex 200 increase 10/300 GL column (Cytiva), equilibrated with 50 mm Tris–HCl pH 8.0 and 150 mm NaCl, to remove free amphipol and assess protein monodispersity.

*Ec*NanX in amphipol was concentrated to 1.65 mg·mL^−1^ and 4 μL of sample was applied to freshly glow‐discharged (Quorum GloQube) Quantifoil R 1.2/1.3400 Cu mesh grids (ProSciTech, Kirwan, QLD, Australia). Grids were blotted using a VitrobotMark IV (Thermo Fisher Scientific) for 4 s, at 4 °C with 100% humidity before vitrification in liquid ethane. Single particle cryo‐electron microscopy (cryo‐EM) datasets were collected on a Titan Krios G4 electron microscope equipped with a K3 detector and BioContinuum imaging filter (Gatan, Pleasanton, CA, USA), operated at 300 kV in counting mode. Movie stacks were collected at a nominal magnification of ×130 000 and a 0.833 Å pixel size. The epu software package (Thermo Fisher Scientific) was used for automated data collection. A total of 5163 micrographs were recorded and the statistics for cryo‐EM data collection are reported in Table S2.

Cryo‐EM data were processed using cryosparc v4.4 [54]. Movie frames were aligned using patch motion correction with a *B‐*factor of 500, and contrast transfer function (CTF) estimations were made using the patch CTF estimation tool. Particles were first picked from 300 micrographs using the Blob Picker tool and extracted. These particles were 2D classified into 100 classes and the best 2D classes were selected and used as a template for automated particle picking using the Template Picker tool. Particles were inspected with the Inspect Picks tool, and a total of 3 574 942 particles were extracted with a box size of 256 pixels. The extracted particles were then sorted using iterative rounds of 2D classification and the best 2D classes resembling monomeric and dimeric *Ec*NanX particles were selected. The selected particles from dimeric classes were subjected to *ab initio* reconstruction and separated into multiple classes. Templates, generated from the best dimer‐like reconstruction, were used for another round of particle picking, before re‐extraction and reclassification. Further *ab initio* reconstructions (~ 12 Å) did not yield viable volumes to continue with refinement and model building. The data collection and processing statistics are reported in Table S2.

### Analytical ultracentrifugation

Sedimentation velocity analytical ultracentrifugation was conducted using a Beckman Coulter Optima analytical ultracentrifuge, with a Beckman Coulter AN50Ti rotor with sapphire windowed cells. *Ec*NanX at 0.5 mg·mL^−1^ (11 μm) was analysed in 50 mm Tris pH 8.0, 150 mm NaCl and 0.02% (w/v) LMNG. Assays were performed at 10 °C, 42 000 rpm, with absorbance (at 290 nm) and interference optics. Sedimentation data were analysed with ultrascan v4.0 [55]. For the absorbance data (290 nm), optimisation was performed by two‐dimensional spectrum analysis (2DSA) [56] with simultaneous removal of time‐ and radially invariant noise contributions and fitting of boundary conditions. Processing was done using a 64 × 64 resolution grid with the bounds of 1–4 for the frictional ratio (*f*/*f*_0_) and 1–15 for the sedimentation coefficient (*S*_(20,W)_).

Following iterative fitting, a parametrically constrained species analysis‐increasing sigmoid (PCSA‐IS) was done to further refine the model. The final solution was generated by subjecting the PCSA‐IS fit to 50 Monte Carlo simulations [57]. Interference data were optimised using a 2DSA with simultaneous removal of time‐ and radially invariant noise contributions and fitting of boundary conditions. This processing was done using a custom grid with resolution of 128 × 128, with 64 × 64 resolution within the boundary of 1–15 *S* and 1–4 *f*/*f*_0_, and 64 × 64 resolution within the boundary of 15–250 *S* and 1–4 *f*/*f*_0_. This was done as the interference optic data had a greater dynamic range than the absorbance optic data, and as such much more signal was collected for minor species present at high *S* values. Following iterative fitting, the model was subjected to parsimonious regularisation by genetic algorithm. The statistics of the final fitted model are reported in Table S3.

To verify the oligomeric state of *Ec*NanX in LMNG, calculations were performed using the membrane protein calculations function of gussi v2.1.6 [58] with buffer density and viscosity measured using a DMA4100M density metre and Lovis 2000 ME viscometer (Anton Paar, Graz, Austria). As UltraScan automatically converts results to *S*_(20,W)_, the buffer density, viscosity, and temperature values were entered into GUSSI as water at 20 °C. The partial specific volume of LMNG (0.797 mL·g^−1^) was used with the sedimentation and diffusion coefficients obtained from UltraScan to calculate the mass of the detergent‐bound *Ec*NanX. The parameters used are reported in Table S4.

### Western blotting

Cell pellets were resuspended in PBS pH 7.4 and normalised to their OD_600_. The samples were then sonicated in a water bath for 5 min, followed by 5 min on ice and another 5 min sonication, before 10 μL of each sample was mixed with 10 μL of 2X Tris‐Glycine SDS sample buffer. After 30 min of incubation, the samples were run using SDS/PAGE, then transferred to a nitrocellulose membrane. The membrane was then washed with 20 mm Tris, 150 mm NaCl and 0.1% (w/v) Tween 20 (TBS‐T), blocked with 5% (w/v) milk powder in TBS‐T for 30 min, before incubation with the anti‐His primary antibody (Invitrogen MA1‐21315, Thermo Fisher Scientific) overnight, with gentle agitation at 4 °C. The membrane was washed before the alkaline phosphatase secondary antibody (Sigma‐Aldrich A1902, Merck KGaA, Darmstadt, Germany) was added and left to incubate with gentle agitation at room temperature for 1 h. Bands were detected with 1 mL 5‐bromo‐4‐chloro‐3′‐indolyphosphate/nitro‐blue tetrazolium (BCIP/NBT) alkaline phosphatase substrate solution.

### *In silico* modelling and substrate docking

alphafold2 [59] models of *Ec*NanT and *Ec*NanX were generated through colabfold [60]. To sample multiple conformations of the transporters [61], a max MSA of 16 : 32 was used, the maximum number of seeds were used, and dropout was selected. To generate dimeric models, alphafold2‐multimer was similarly used through colabfold. autodock vina v1.2.5 (part of mgltools v1.5.7) [62] was used to dock Neu5Ac and 2,7‐anhydro‐Neu5Ac into the models of *Ec*NanT and *Ec*NanX. Kollman charges and polar hydrogen atoms were added to both models and ligands to prepare them. The grid box was placed tight to the substrate‐binding site and an energy range of 4 kcal·mol^−1^ was used. Chai‐1 [63] was used with no MSAs, templates or restraints to predict the 3D structures of substrate bound NanX and NanT. Chai‐1 was accessed via the web server (https://www.chaidiscovery.com/).

### Multiple sequence alignment

The protein sequences used in the multiple sequence alignment are from major facilitator superfamily (MFS) transporters associated with sialic acid catabolic (*nan*) genes, as identified by Severi *et al*., [42]. Sequences were obtained from the National Center for Biotechnology Information, aligned using clustal omega [64] and coloured by clustal x colouring in Jalview [65]. The species name and accession code of each sequence is listed in Table S5.

### Bacterial growth assay

pJ422_*EcnanT*, pJ422_*EcnanX* and mutant constructs were transformed into the *E. coli* JW3193 Δ*nanT* strain. A single colony of each strain was grown overnight in Luria broth (LB) medium (all media were supplemented with 25 μg·mL^−1^ Zeocin throughout) at 37 °C, shaking at 180 rpm. The overnight cultures were used to inoculate fresh cultures of LB medium, supplemented with 1 mm IPTG to induce expression of the transporters. These cultures were grown to an OD_600_ of 0.35 before being washed three times in M9 minimal medium and resuspended at an OD_600_ of 0.5. 20 μL of the resuspended cells was added to 180 μL of M9 minimal medium, containing 1 mm IPTG, 7 μm thiamine hydrochloride and either 12.9 mm Neu5Ac, 0.4% (w/v) glucose or no carbon source. Growth at 37 °C with shaking at 300 rpm was monitored by OD_600_ every 10 min using a FLUOstar Omega Microplate Reader (BMG Labtech, Ortenberg, Germany). *E. coli* JW3193 Δ*nanT* both untransformed and transformed with an empty vector (pJ422_empty) were included as controls. To account for any residual LB medium, data were analysed and are shown from the 2‐h time point, normalised to an initial OD_600_ of 0.05. Technical triplicates (i.e. three wells) were used in the initial experiment. A second experiment with newly transformed bacteria was also performed with technical duplicates.

## Results and Discussion

### Purification of Ec*NanX*

**Fig. 2:**
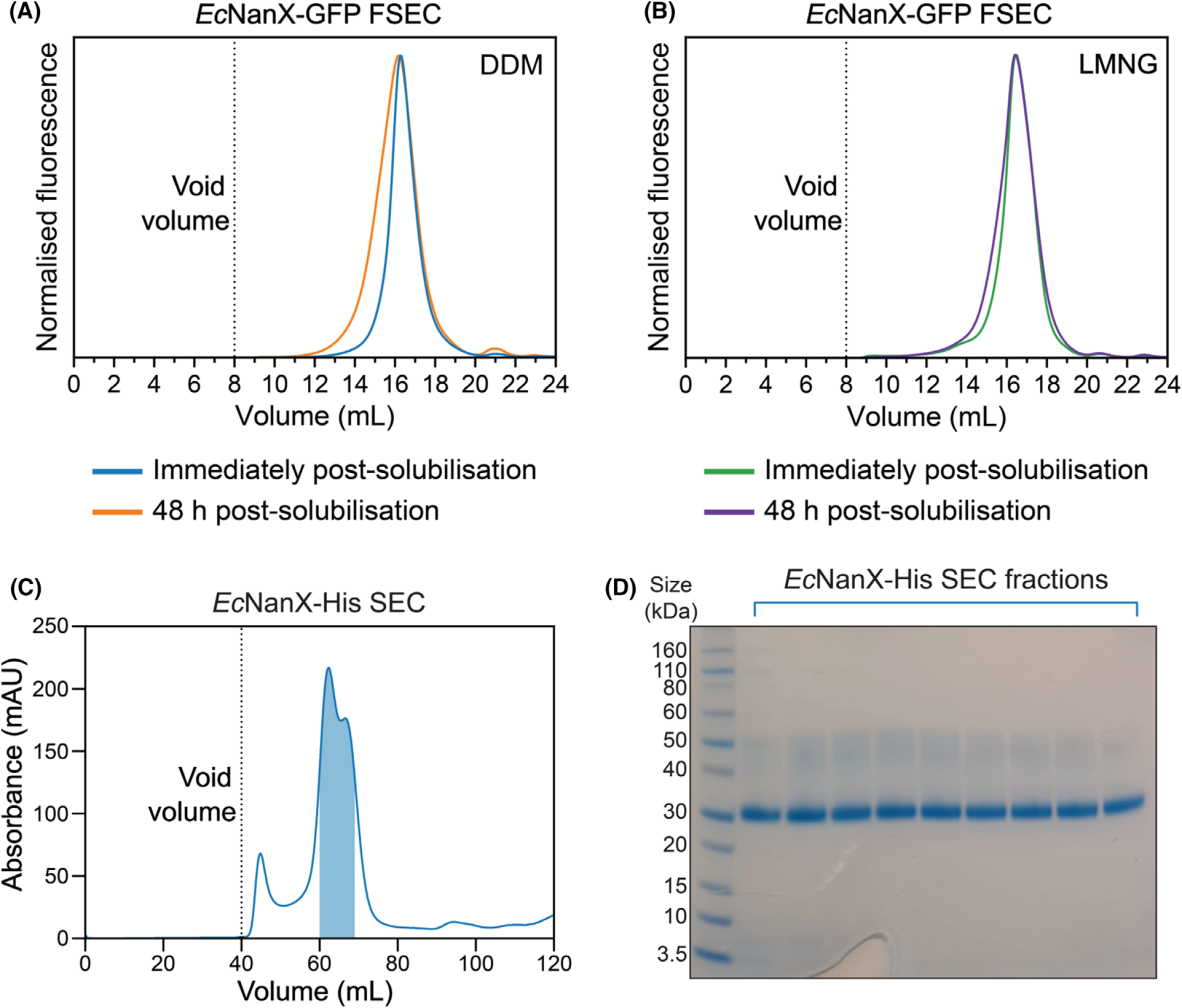
Detergent screening and purification of *Ec*NanX. (A) *Ec*NanX‐GFP was stabilised by n‐dodecyl‐β‐*d*‐maltopyranoside (DDM) immediately following membrane solubilisation (blue trace) and after 48 h at 4 °C (orange trace). (B) *Ec*NanX‐GFP was also stabilised by lauryl maltose neopentyl glycol (LMNG) immediately following solubilisation (green trace) and after 48 h at 4 °C (purple trace). (C) Size‐exclusion chromatography (SEC) elution profile of LMNG‐extracted *Ec*NanX‐His, the area shaded blue (~ 60–70 mL elution volume) corresponds to the fractions that were analysed with SDS/PAGE. (D) SDS/PAGE analysis of the SEC elution fractions between ~ 60–70 mL (shaded blue in panel C).

We investigated *Ec*NanX‐GFP stability in different detergents using fluorescence size‐exclusion chromatography (FSEC). *Ec*NanX‐GFP was stable in both n‐dodecyl‐β‐*d*‐maltoside (DDM) and lauryl maltose neopentyl glycol (LMNG). To evaluate whether *Ec*NanX‐GFP was stable over time, we incubated the detergent‐solubilised membrane samples at 4 °C for 48 h and then compared their elution profiles. After 48 h, the elution profile of DDM broadened, but remained largely symmetrical (Fig. 2A). The sample in LMNG displayed subtle broadening after 48 h, indicating that both detergents effectively stabilised *Ec*NanX‐GFP (Fig. 2B).

To simplify the purification, we used a C‐terminally His‐tagged *Ec*NanX construct. *Ec*NanX‐His was purified using LMNG as the stabilising detergent. The SEC elution profile displayed a minor peak at the void volume of the column (approximately 45 mL, Fig. 2C). *Ec*NanX primarily eluted between 60 and 70 mL with a pronounced shoulder off the right side of the main peak (Fig. 2C). SDS/PAGE analysis of the 60–70 mL region revealed a minor contaminating protein at ~ 50 kDa (Fig. 2D). We hypothesised that the double SEC peak along with the secondary band in SDS/PAGE could represent the formation of a higher order *Ec*NanX species. This is supported through the literature since some MFS transporters function as dimers in their native membrane environment while others dimerise under purification conditions [66, 67]. However, the SEC of purified *Ec*NanX‐His should be compared to the FSEC experiment, in which *Ec*NanX‐GFP directly solubilised from membranes elutes in a single stoichiometric state (Fig. 2A,B). While the FSEC indicates neither monomer nor dimer, but simply a single species, it does diverge from observations made in the purified *Ec*NanX‐His SEC experiment and further biophysical experiments herein. The discrepancy in the traces could be a product of several factors: (a) *Ec*NanX‐His dimer formation during purification, (b) an artefact of the GFP tag on *Ec*NanX‐GFP, or (c) a concentration effect, as the concentration of *Ec*NanX‐GFP is likely to be much lower than that of purified *Ec*NanX‐His.

### Oligomeric state of Ec*NanX*

**Fig. 3:**
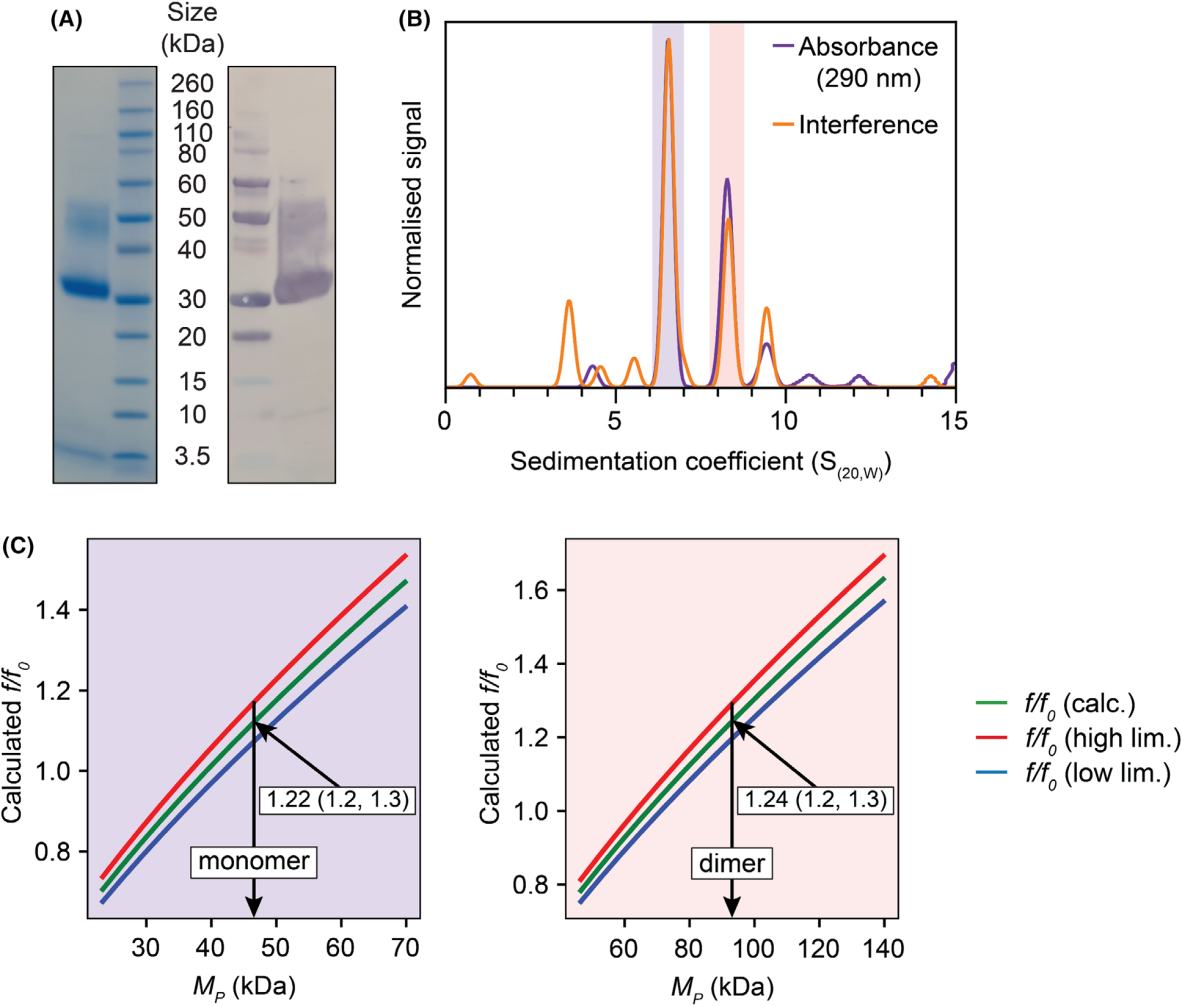
Assessing the oligomeric state of *Ec*NanX. (A) Western blot analysis of purified *Ec*NanX. An anti‐His primary antibody was used with an alkaline phosphatase secondary antibody and BCIP/NBT substrate to detect the two *Ec*NanX bands. (B) Analytical ultracentrifugation analysis using absorbance (290 nm, purple) and interference (orange) optics. (C) The oligomeric state of the major *Ec*NanX species was calculated using the membrane protein calculation function in gussi [58], with plots showing the calculated frictional ratio (*f*/*f*_0_) corresponding to the oligomeric state for the *Ec*NanX species at 6.6 S (purple, left) and 8.3 S (pink, right). The analytical ultracentrifugation statistics and parameters used for the oligomeric state calculations are reported in Tables S3 and S4, respectively.

Western blot analysis could suggest that *Ec*NanX forms an oligomer due to the presence of higher molecular weight signal, however given the propensity of membrane proteins to display aberrant migration patterns in SDS/PAGE [68], this result should be viewed with caution (Fig. 3A). To gain higher resolution information, we conducted a sedimentation velocity analytical ultracentrifugation experiment, in which *Ec*NanX sedimented as two main species (Fig. 3B) with sedimentation coefficients (*S*_(20,W)_) of 6.6 *S* and 8.3 *S* (~ 49% and 32% of the absorbance signal, respectively). Another peak at just under 4 *S* likely corresponds to empty LMNG micelles and explains why it is only present in the interference data. To confirm the oligomeric state of the two major *Ec*NanX species, we utilised the membrane protein calculation function in GUSSI (Fig. 3C) [58]. In short, data from absorbance and interference optics enable quantification of the protein and detergent components for each peak. Based on the relative ratios for each component, one can then determine the most reasonable oligomeric conformation that would satisfy the experimental data. In‐depth discussions of the technique are available in the work of Brautigam that expands upon the work of Ebel and others [58, 69, 70]. The first peak at 6.6 *S* corresponds to a monomeric *Ec*NanX at ~ 46.5 kDa, complexed with ~ 104 molecules of LMNG. The second peak at 8.3 *S* corresponds to a dimeric *Ec*NanX species complexed with ~ 119 molecules of LMNG. These observations are consistent with expectations for the number of complexed detergent molecules previously seen in proteins of similar sizes by ourselves and others [29, 48]. The peaks greater than ~ 9 *S* do not correspond to a discrete oligomeric *Ec*NanX species based on the gussi calculations (Fig. S1) and are likely due to the formation of aggregates over the course of the experiment. This matches SEC observations, where two discrete species were present in addition to aggregates (Fig. 2C). While the results do confirm the presence of an *Ec*NanX dimer after purification, they do not assert a physiological relevance to the higher order oligomer.

Having confirmed the presence of both *Ec*NanX monomers and dimers in our purified preparation, we reasoned that *Ec*NanX could be amenable to structural determination using single‐particle cryo‐electron microscopy (cryo‐EM) because of the increased size of the dimeric species. Purified *Ec*NanX was exchanged into amphipols (Fig. S2A) and then used to prepare cryo‐EM grids. Cryo‐EM data were collected (Fig. S2B), and initial 2D classification clearly showed the presence of both *Ec*NanX monomers and dimers (Fig. S2C,D). However, further data processing of the dimeric classes (not attempted with the monomer, given its smaller size and less detailed 2D classes) did not yield a high‐resolution 3D reconstruction (Fig. S2C). The final 3D reconstruction of the dimer was consistent with the elongated 2D classes, although no defining features outside of the amphipol layer were revealed. Given the small size of the *Ec*NanX dimer and the absence of distinguishing features outside the amphipol layer, a fiducial marker protein, such as a nanobody, may be required to resolve a high‐resolution structure in future. Nonetheless, these results are encouraging for future efforts given the clear 2D classes and low‐resolution 3D reconstruction.

### *In silico* modelling of *Ec* NanX and *Ec* NanT

**Fig. 4:**
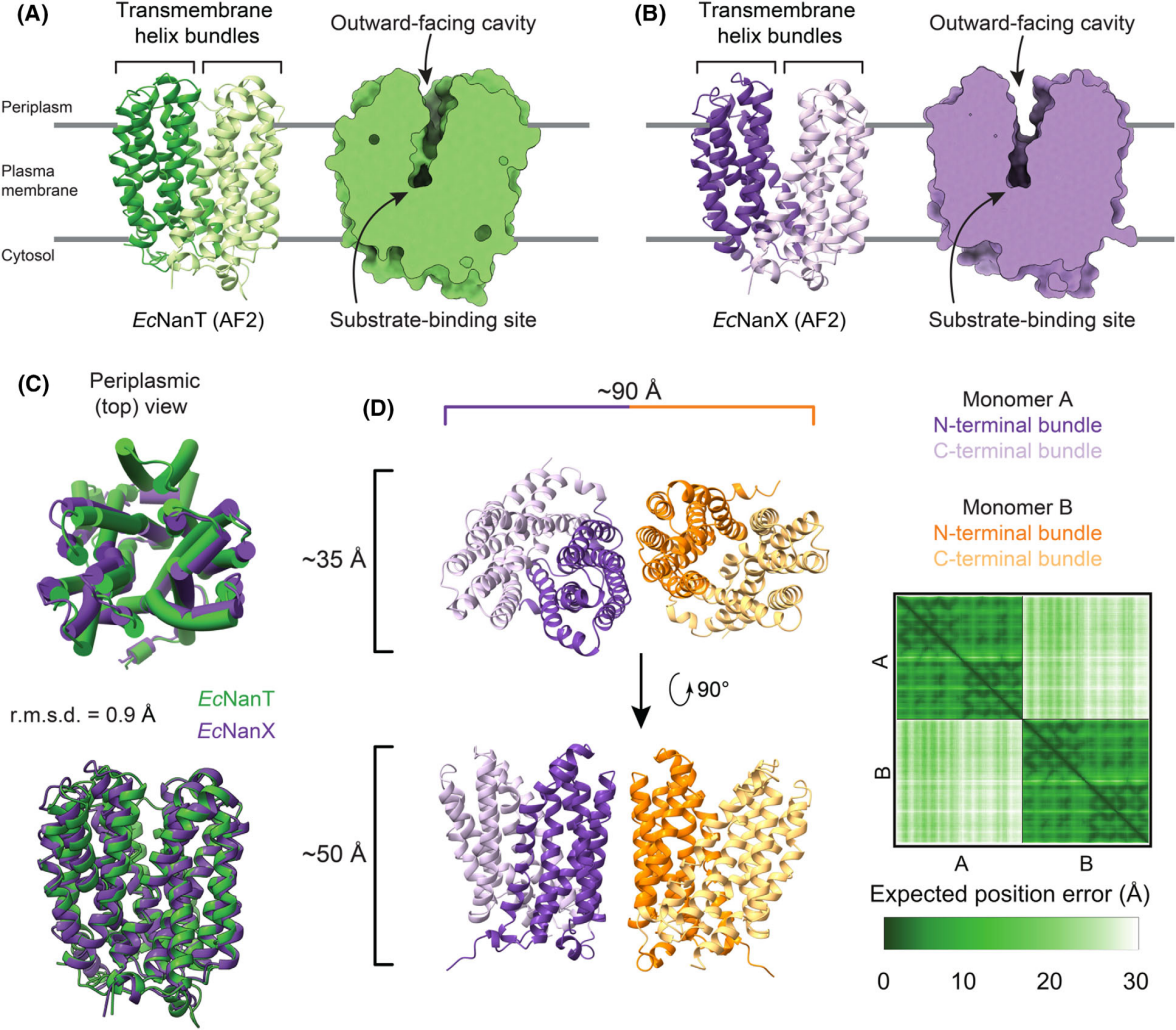
Modelling the structures of *Ec*NanT and *Ec*NanX using alphafold2 [59]. (A) The model of *Ec*NanT comprises two bundles of seven transmembrane helices (left), with a central cutaway revealing the substrate‐binding site and the outward‐facing (open to the periplasm) conformation (right). (B) The model of *Ec*NanX comprises two bundles of six transmembrane helices (left), with a central cutaway revealing the translocation pathway and the outward‐facing (open to the periplasm) conformation (right). (C) Structural overlay of *Ec*NanT and *Ec*NanX models, which align with a root‐mean‐squared deviation (r.m.s.d.) of 0.9 Å across 295 α‐carbons. For visual clarity, the residues modelled with low confidence at each terminus were removed. (D) The low probability dimeric alphafold2 model of *Ec*NanX (see the high expected position error between protein chains within the predicted aligned error plot) has a parallel arrangement and is consistent in size and shape with the low resolution cryo‐EM data (Fig. S2). Models are visualised using chimerax [77].

Unable to refine a high‐resolution cryo‐EM structure, we used alphafold2 [59] to generate structural models of both *Ec*NanX and *Ec*NanT (Fig. 4, confidence metrics are presented in Fig. S3). Consistent with previous topology predictions [38], the model of *Ec*NanT revealed two bundles of seven transmembrane helices (14 total) in the outward‐facing open conformation, with the substrate‐binding site formed between them (Fig. 4A). In line with the typical architecture of MFS transporters, *Ec*NanX was predicted to contain two bundles of six transmembrane helices (12 total) also in the outward‐facing open conformation, with its substrate‐binding site located between the bundles (Fig. 4B). A structural alignment highlighted their conserved architecture, aside from the two additional transmembrane helices in *Ec*NanT (Fig. 4C).

Our AUC and low‐resolution cryo‐EM results demonstrate that *Ec*NanX forms both monomers and dimers, so we generated models of an *Ec*NanX dimer using alphafold2‐multimer [59] to assess potential dimeric interfaces and orientations. alphafold2 confidence metrics for the predicted dimeric structure are low, with interface predicted template modelling (ipTM) scores of 0.141–0.263. alphafold2‐multimer will generate an oligomer regardless of biological relevance, indeed alphafold2 also generated low confidence (ipTM 0.147–0.469) dimeric models of *Ec*NanT (Fig. S3B). Whether *Ec*NanT similarly forms a dimer is unknown. The dimeric model presented in Fig. 4D (ipTM 0.261) has a buried surface are of ~ 1700 Å^2^ [71] and dimensions that are consistent with the low‐resolution cryo‐EM data (Fig. S2). The larger volume of the cryo‐EM map represents extra density from the amphipol layer surrounding *Ec*NanX that is not present in the model (Fig. S2C). The amalgamated data do not prove that *Ec*NanX adopts a dimeric conformation under native conditions, but our observation of a dimeric species could help in future structural studies of the transporter.

**Fig. 5:**
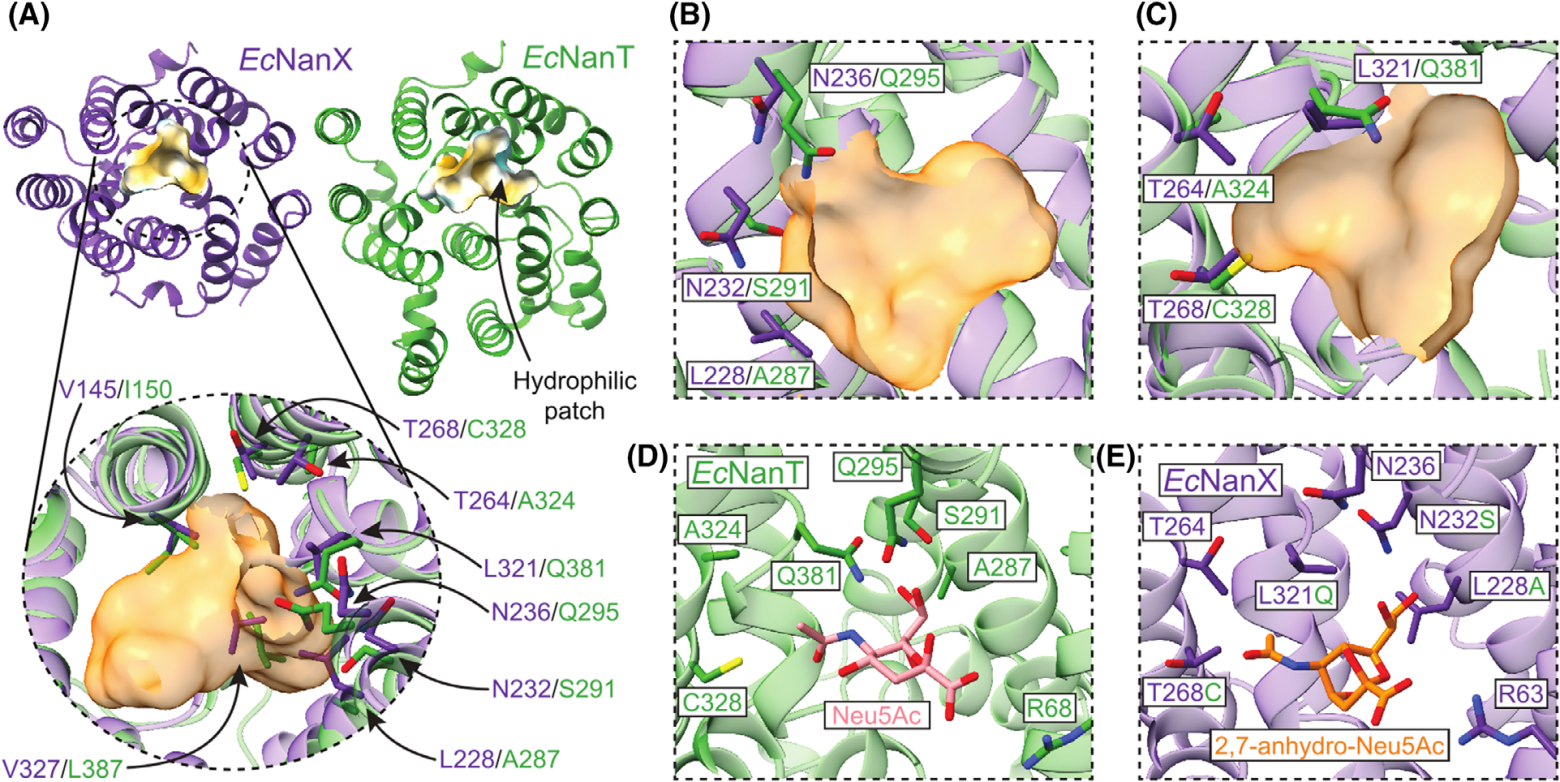
Substrate‐binding site comparison of *Ec*NanX and *Ec*NanT. (A) alphafold2 predicted substrate‐binding site comparison of *Ec*NanX (left, purple) and *Ec*NanT (right, green). Residues differing between the substrate‐binding sites are highlighted in the zoomed inset below. Only the substrate‐binding site cavity of *Ec*NanX is shown for clarity (orange surface). (B) First set of significantly differing substrate‐binding site residues between *Ec*NanX (purple) and *Ec*NanT (green). The substrate‐binding site cavity of *Ec*NanX is shown (orange surface). (C) Second set of significantly differing substrate‐binding site residues between *Ec*NanX (purple) and *Ec*NanT (green). The substrate‐binding site cavity of *Ec*NanT is shown (orange surface). (D) Docking *N*‐acetylneuraminate (Neu5Ac) into the model of *Ec*NanT. (E) Docking 2,7‐anhydro‐*n*‐acetylneuraminate (2,7‐anhydro‐Neu5Ac) into the model of *Ec*NanX. Differing substrate‐binding site residues are highlighted, with the mutations marked in green (L228A, N232S, T268C and L321Q). A double *Ec*NanX mutant was also constructed, L228A/L321Q. Models are visualised using chimerax [77].

Initial alphafold2 models displayed outward facing conformations of *Ec*NanX and *Ec*NanT. To analyse the substrate‐binding sites, we restrained the MSA to increase model heterogeneity and access additional conformations of the transporters [61]. While this method does not take into consideration the potential impact of substrate binding on conformational transitions, it nonetheless generated models in an occluded state, where substrate‐binding sites are inaccessible to both periplasm and cytoplasm (Fig. 5, confidence metrics are presented in Fig. S3). The putative substrate‐binding site of each transporter is located between the two helical bundles and is consistent in size for the binding of a small sugar molecule (Fig. 5A). The main difference observed in this conformation is the presence of a hydrophilic region in the *Ec*NanT substrate‐binding site (indicated on the right‐hand side of the binding site surface in Fig. 5A). While most substrate‐binding site residues are conserved between both transporters, several residues differed (Fig. 5B,C).

### Identification and functional characterisation of substrate specific residues in *Ec*NanX and *Ec*NanT

To assess the importance and potential function of the differing residues within the substrate‐binding sites of *Ec*NanX and *Ec*NanT, we conducted a multiple sequence alignment of 25 transporters homologous to *Ec*NanX or *Ec*NanT and performed *in silico* docking with their respective substrates. The multiple sequence alignment identifies a conserved arginine across all species sampled (R63 in *Ec*NanX and R68 in *Ec*NanT). In other sialic acid transporters and binding proteins, arginine residues have known roles in coordinating sialic acid carboxylate groups [18, 35, 36, 72, 73]. Using this knowledge, we focused on the docking results where the substrate carboxylate group was oriented towards this arginine residue (Fig. 5D,E). Upon docking of either Neu5Ac or 2,7‐anhydro‐Neu5Ac into *Ec*NanT or *Ec*NanX, respectively, so that the carboxylates are oriented towards the conserved arginine, the substrates' acetyl groups and glycerol tails adopt a position oriented towards regions less conserved between NanX and NanT homologues, potentially conferring specificity. While less conserved between NanX and NanT homologues, this patch of residues adjacent to the substrate‐binding site is nonetheless conserved across homologues for each individual transporter. In other words, NanT homologues have conserved set of residues, as do NanX homologues, but these sets differ across the two transporters (Figs 5B and S4). This suggests that these residues may have a role in differentiating Neu5Ac and 2,7‐anhydro‐Neu5Ac. Specifically, a hydrophobic residue is conserved among NanX homologues (L228 in *Ec*NanX), while the NanT homologues predominantly have a considerably smaller alanine or serine residue at the equivalent position (A287 in *Ec*NanT). Adjacent to this, an asparagine residue (N232 in *Ec*NanX) is universally conserved across NanX homologues, while NanT transporters typically have a conserved serine or threonine residue at the equivalent position (S291 in *Ec*NanT).

Nearby, T264 in *Ec*NanX is partially conserved, while NanT homologues at the corresponding position (A324 in *Ec*NanT) have an alanine or asparagine residue, suggesting a role in substrate specificity, although some residue variability is evident in both NanX and NanT homologues (Figs 5C and S4). T268 in *Ec*NanX is largely conserved among NanX homologues, while cysteine or tyrosine residues are present at the equivalent position in NanT homologues (C328 in *Ec*NanT). Finally, a substantial difference is observed at position L321 in *Ec*NanX where NanX homologues have a conserved hydrophobic residue, while NanT homologues have an invariant glutamine (Q381 in *Ec*NanT) at the equivalent position (Fig. S4).

The docking results suggest that the *N*‐acetyl and glycerol tail groups, where Neu5Ac and 2,7‐anhydro‐Neu5Ac differ structurally, are directed into the regions where the *Ec*NanT and *Ec*NanX substrate‐binding sites diverge (Fig. 5D,E), consistent with the arginine‐carboxylate mediated interaction model. We also used Chai‐1 to predict 3D structures of *Ec*NanX and *Ec*NanT with their respective substrates and the results largely coincide with the with the data shown in Fig. 5, including carboxylate orientation towards the conserved arginine (Fig. S5).

To validate the involvement of purported key residues identified in the multiple sequence alignment and *in silico* substrate docking experiments, we designed four single mutants and one double mutant of *Ec*NanX. The mutants were designed to alter the substrate specificity of *Ec*NanX and enable it to transport the *Ec*NanT‐specific substrate, Neu5Ac. The mutants included: *Ec*NanX^L228A^, *Ec*NanX^T268C^, *Ec*NanX^N232S^, *Ec*NanX^L321Q^ and *Ec*NanX^L228A/L321Q^. Rationale for the mutants is presented in Table 1.

**Table 1:** Rationale for site‐directed mutagenesis.

Mutant	Rationale
*Ec*NanX^L228A^	Conserved residues at this position, mutation may allow more space for the Neu5Ac glycerol moiety to occupy
*Ec*NanX^T268C^	Semi‐conserved residues, removing a methyl group may enable the *N*‐acetyl group of Neu5Ac to be positioned differently to 2,7‐anhydro‐Neu5Ac
*Ec*NanX^N232S^	Maintains a hydrogen bonding opportunity but also provides more space for the Neu5Ac glycerol moiety to occupy
*Ec*NanX^L321Q^	Universally conserved residues, the introduction of a polar residue may provide hydrogen bonding opportunities for the Neu5Ac glycerol moiety
*Ec*NanX^L228A/L321Q^	May act cooperatively to coordinate the Neu5Ac glycerol tail in *Ec*NanT

**Fig. 6:**
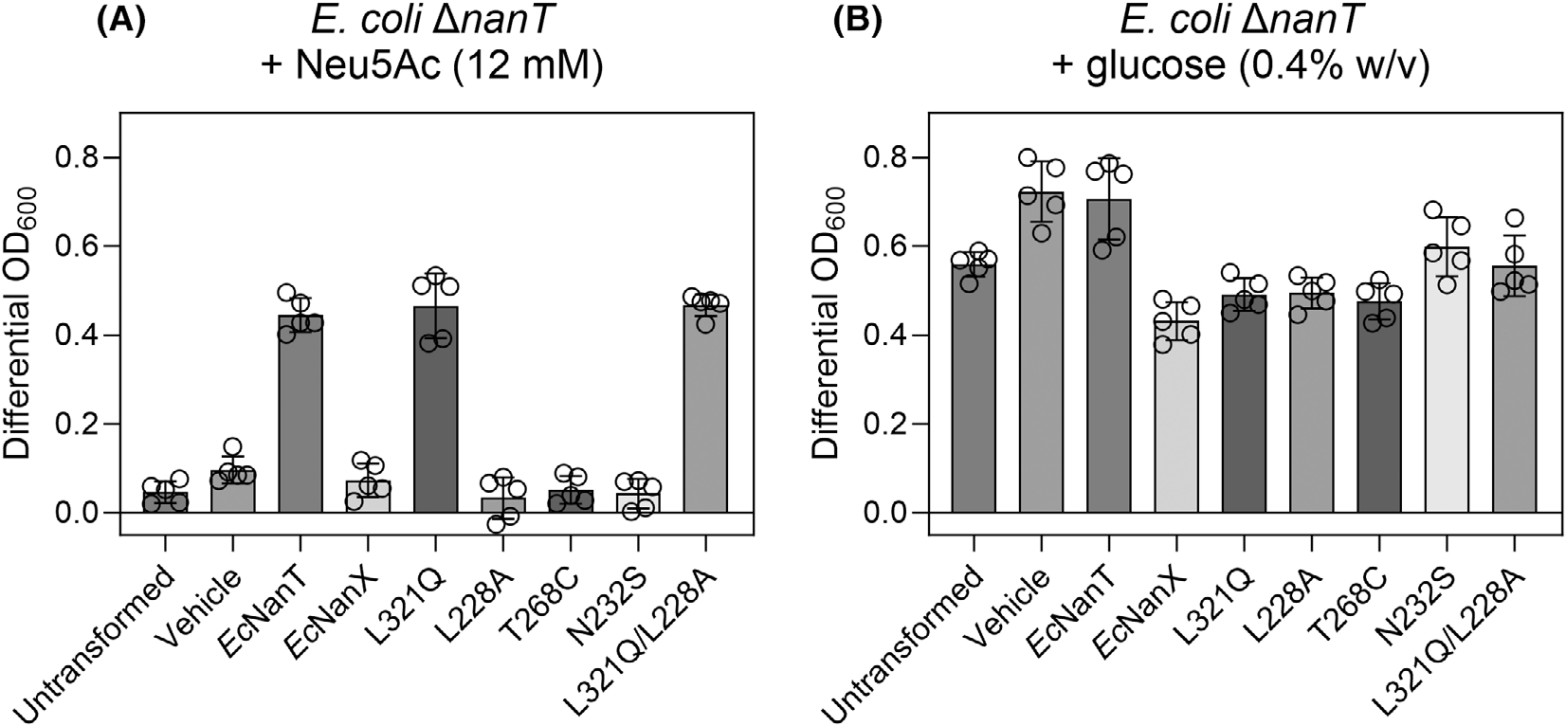
Bacterial growth experiments demonstrate *N*‐acetylneuraminate (Neu5Ac) transport by *Ec*NanX mutants. *E. coli* Δ*nanT*, untransformed or transformed with empty plasmid (vehicle), *Ec*NanT, *Ec*NanX, or *Ec*NanX mutants, were grown in M9 minimal medium supplemented with Neu5Ac (12.9 mm) or glucose (0.4% w/v) as the sole carbon source. Growth after 50 h is shown as differential OD_600_; that is, the difference in OD_600_ at start and end timepoints. Data are presented as mean values with standard deviations across five technical replicates from two independent experiments. (A) Differential growth of the *E. coli* strains in Neu5Ac‐supplemented minimal medium. (B) Growth of the *E. coli* strains with glucose as the carbon source. Growth curves across 50 h are shown in Fig. S6.

We used a bacterial growth assay and a Δ*nanT* knockout *E. coli* strain to gauge whether the *Ec*NanX mutants altered substrate specificity. Untransformed Δ*nanT* bacteria are unable to grow in media where Neu5Ac is the sole carbon source (Figs 6A and S6A). Growth of the Δ*nanT* bacteria transformed with mutant *Ec*NanX constructs (Table 1) and controls in the presence of Neu5Ac or glucose were measured to determine transporter substrate specificity.

When compared to the untransformed Δ*nanT* strain and vehicle control, Δ*nanT* bacteria transformed with constructs encoding *Ec*NanT, *Ec*NanX^L321Q^ or *Ec*NanX^L228A/L321Q^ showed growth over 50 h (Figs 6A and S6A). WT *Ec*NanX or other *Ec*NanX mutants had no effect on bacterial growth (Figs 6A and S6A). As expected, all strains grew over 50 h in the presence of glucose (Figs 6B and S6B), and none grew in the absence of a carbon source (Fig. S6E).

It is unsurprising that transformation with *Ec*NanT yields growth in *E. coli* Δ*nanT* as it restores the physiological Neu5Ac transporter. *Ec*NanX^L228A/L321Q^‐ and *Ec*NanX^L321Q^‐mediated growth demonstrate the importance of the equivalent glutamine residue in *Ec*NanT (Q381) for Neu5Ac transport. It is possible that the Q381 forms hydrogen bonds with the hydroxyl head groups of the glycerol side chain of Neu5Ac extended in its direction, as suggested by the docking results (Figs 5 and S5). As C7 of 2,7‐anhydro‐Neu5Ac indeed takes part in the 2,7‐anhydro bridge to C2, the glycerol side chain has a more compact conformation when compared with Neu5Ac. It is likely not extending towards the side chains of helix 10 of NanX (helix 12 in NanT) that partially lines the substrate‐binding pocket and harbours L321 in NanX (Q381 in NanT) (Fig. S4). These results suggest that hydrogen bonding potential that can accommodate the more flexible and extended glycerol side chain of Neu5Ac is important for its transport.

Transformation of *E. coli* Δ*nanT* with *Ec*NanX^L228A^ does not restore growth as measured in our assay; however, this amino acid substitution does not hinder growth when combined with *Ec*NanX^L321Q^ (Figs 6 and S6). While the L228A substitution may increase the volume of the binding pocket for the extended glycerol tail of Neu5Ac to occupy, it does not introduce any hydrogen bonding opportunities to preferentially interact with the hydrogen head groups of Neu5Ac's glycerol tail. Although the equivalent position in NanT (A287) neither participates in hydrogen binding, this potential requirement may be carried out by Q381, as we commented on earlier. Again, this emphasises the importance of L321Q in likely providing the hydrogen binding opportunities at the Neu5Ac glycerol moiety, while the benign L228A is sterically accommodating.

## Conclusion

Our study aimed to unravel the mechanism of sialic acid import in *E. coli*, mediated by the MFS transporters *Ec*NanX and *Ec*NanT. Sialic acids are crucial in the colonisation and persistence of many pathogenic bacteria [42, 74], and understanding these processes may aid in the development of novel antibiotics. We report conditions for the successful purification of the 2,7‐anhydro‐Neu5Ac MFS transporter, *Ec*NanX. While we ascribe no physiological importance to the observed oligomeric state, we show that purified *Ec*NanX exists as a monomer and dimer, and we demonstrate that the protein is tractable for structural determination by cryo‐EM. However, it may prove useful to develop fiducial marker proteins specific to *Ec*NanX to increase the particle size and aid in alignment; MFS transporters elsewhere have been structurally characterised using nanobodies or Fab fragments [75, 76].

To further investigate the molecular basis of substrate specificity, we generated alphafold2 models of both *Ec*NanX and *Ec*NanT and examined their substrate‐binding site residues. Structural modelling, substrate docking and residue conservation analysis identified key amino acids that might determine substrate specificity. Guided by these insights, site‐directed mutants of *Ec*NanX were produced and transport activity was assessed using a bacterial growth assay, identifying residues involved in substrate transport. Further work to build on this includes addressing the following questions: do the reported *Ec*NanX amino acid substitutions transfer specificity for 2,7‐anhydro‐Neu5Ac to Neu5Ac or rather decrease selectivity of the transporter regardless of substrate? Our study has not explored whether we conferred specificity for Neu5Ac through the mutations made or simply made NanX a more promiscuous transporter. Neither have we made reciprocal substitutions in *Ec*NanT to test whether they confer the ability to transport 2‐7‐anhydro‐Neu5Ac and how they affect transport of *Ec*NanT's native substrate.

Collectively, our findings provide a framework for further structural and functional studies of *Ec*NanX and *Ec*NanT. Given that sialic acid uptake is a clinically relevant pathway for pathogenic bacteria [49], understanding the mechanisms that underpin selective transport of 2,7‐anhydro‐Neu5Ac and Neu5Ac may prove valuable for antibiotic development and warrants further work to flesh out transport details of both compounds.

## Conflict of interest

The authors declare no conflicts of interest.

## Author contributions

MCN‐V was involved in conceptualisation, formal analysis, investigation, methodology, visualisation, writing—original draft, review & editing, finalising manuscript. KRH and ZDT were involved in formal analysis, investigation, methodology. LST was involved in formal analysis, review & editing, finalising manuscript. JSD and MJC were involved in conceptualisation, resources, validation, supervision, review & editing. HGB, SV, PDM, AEW, SP and EH were involved in formal analysis, investigation, methodology. RAN was involved in conceptualisation, resources, supervision, writing—review & editing. RCJD was involved in conceptualisation, funding acquisition, project administration, resources, supervision, writing*—*review & editing.

## Supporting information


**Fig. S1.** Membrane protein calculations on the additional species observed in the analytical ultracentrifugation experiments with *Ec*NanX.
**Fig. S2.** Cryo‐EM screening of *Ec*NanX.
**Fig. S3.** Confidence metrics of the *Ec*NanX and *Ec*NanT models used in this study.
**Fig. S4.** Multiple sequence alignment of the NanX and NanT homologues identified by Severi *et al*., [42].
**Fig. S5.** Comparison of substrate binding using *in silico* methods.
**Fig. S6.** Bacterial growth assay.
**Table S1.** Primer sequences used in this study.
**Table S2.** Cryo‐EM data collection statistics for recombinant *Ec*NanX.
**Table S3.** Sedimentation velocity analytical ultracentrifugation analysis of *Ec*NanX.
**Table S4.** Parameters used to determine the oligomeric state of *Ec*NanX with analytical ultracentrifugation.
**Table S5.** The NanX and NanT homologues used in the multiple sequence alignment.

## Data Availability

The data that support the findings of this study are available from the corresponding author renwick.dobson@canterbury.ac.nz upon reasonable request.
