# Supplementary material for: Structural studies and functional engineering of NanX: an anhydro‐sialic acid transporter from Escherichia coli
Source: FEBS Open Bio. 2026 Jul 14:10.1002/2211-5463.70310. Online ahead of print. doi: 10.1002/2211-5463.70310 (PMC13398819; doi:10.1002/2211-5463.70310)
Supplement: Supplementary file 1 — Fig. S1. Membrane protein calculations on the additional species observed in the analytical ultracentrifugation experiments with EcNanX. Fig. S2. Cryo‐EM screening of EcNanX. Fig. S3. Confidence metrics of the EcNanX and EcNanT models used in this study. Fig. S4. Multiple sequence alignment of the NanX and NanT homologues identified by Severi et al., [42]. Fig. S5. Comparison of substrate binding using in silico methods. Fig. S6. Bacterial growth assay. Table S1. Primer sequences used in this study. Table S2. Cryo‐EM data collection statistics for recombinant EcNanX. Table S3. Sedimentation velocity analytical ultracentrifugation analysis of EcNanX. Table S4. Parameters used to determine the oligomeric state of EcNanX with analytical ultracentrifugation. Table S5. The NanX and NanT homologues used in the multiple sequence alignment. [file FEB4-9999-0-s001.docx]

**Table S1 | Primer sequences used in this study.**

| **Primer** | **Sequence** |
| --- | --- |
| *EcnanX*_fwd | AGACCTTAGGAGGTAAACATATGGCAACAGCTTGGTATAAACAA |
| *EcnanX*_rev | TGCAGGCCTGTACAGAATTCTTATTTCGCGACGGACAGCTTGTAGAT |
| pJ422_fwd | GAATTCTGTACAGGCCTGCAGAGA |
| pJ422_rev | ATGTTTACCTCCTAAGGTCTCGAA |
| L228A_fwd | GTTTTTGTGCGTTTGGTGCAAATTGGCCTATTAATGGTCTGCT |
| L228A_rev | CACCAAACGCACAAAAACACACCAGGAACACGATCATGGAGAT |
| N232S_fwd | TTGGTGCATCTTGGCCTATTAATGGTCTGCTGCCGTCCTATCT |
| N232S_rev | TAGGCCAAGATGCACCAAACAGACAAAAACACACCAGGAACAC |
| T268C_fwd | CCGGGTGTATTTTCTTCGGCTTTGTTGGTGATAAGATCGGCGT |
| T268C_rev | CGAAGAAAATACACCCGGTCAACGTGCCGAGACCCGCAATGGT |
| L321Q_fwd | TACCAACCAGGGGATCGCGGGTCTTGTGCCGAAGTTTATTTAC |
| L321Q_rev | GCGATCCCCTGGTTGGTAAACATCAGACCGAACAAGCACAAAC |

**Table S2 | Cryo-EM data collection statistics for recombinant *Ec*NanX.**

| **Data collection and processing** |  |
| --- | --- |
| Magnification | 130,000 |
| Voltage (kV) | 300 |
| Electron exposure (e^–^/Å^2^) | 66 |
| Defocus range (μm) | -2.0 to -0.8 |
| Pixel size (Å) | 0.833 |
| Symmetry imposed |  |
| Initial particle images (no.) | 3,574,942 |
| Final particle images (no.) | 73,487 |
| Map resolution (Å) | 11.8 |
| FSC threshold | 0.143 |

**Table S3 | Sedimentation velocity analytical ultracentrifugation analysis of *Ec*NanX.** *Ec*NanX was used at 0.5 mg/mL (11 μM) and has a molecular weight calculated from the amino acid sequence of 46.5 kDa. The data was analysed using UltraScan v4.0 [1] and is presented as plotted in **Figures 3B** and **S6**.

| **Optics** | **Peak S_20,w_ (S)** | **Proportion of the signal (%)** | ***f/f_o_*** | **Fitted mass (kDa)** | **Variance** | **r.m.s.d.** |
| --- | --- | --- | --- | --- | --- | --- |
| Absorbance (290 nm) | 6.55 | 48.69 | 1.26 | 130.5 | 8.08e^-06^ | 0.00284 |
|  | 8.27 | 31.75 | 1.41 | 220.9 |  |  |
|  | 9.43 | 7.58 | 1.55 | 308.5 |  |  |
| Interference | 6.60 | 43.90 | 1.48 | 180.7 | 8.23e^-05^ | 0.00907 |
|  | 8.32 | 19.86 | 1.49 | 240.5 |  |  |
|  | 9.44 | 9.38 | 1.01 | 361.6 |  |  |

**Table S4 | Parameters used to determine the oligomeric state of *Ec*NanX with analytical ultracentrifugation.** The calculations were completed in GUSSI [2], (P) = protein, (D) = detergent (LMNG).

| **Parameters** | ***Ec*NanX in LMNG** |
| --- | --- |
| Buffer density (g/mL) | 0.99823 |
| Buffer viscosity (poise) | 0.01002 |
| Temperature (K) | 283.15 |
| Laser wavelength (nm) | 660.0 |
| Mass extinction coefficient (P, l/g.cm at 290 nm) | 1.047 |
| Partial specific volume (P, mL/g) | 0.756 |
| Molecular mass (P, Da) | 46,547.10 |
| Refractive index increment (P, mL/g) | 0.187 |
| Partial specific volume (D, mL/g) | 0.797 |
| Molecular mass (D, Da) | 1,005.2 |
| Refractive index increment (D, mL/g) | 0.146 |

**Table S5 | The NanX and NanT homologues used in the multiple sequence alignment.** The abbreviation used in the multiple sequence alignment (**Figure S3**) is shown with the corresponding sequence’s species name and GenBank accession.

| **Abbreviation** | **Species** | **GenBank accession** |
| --- | --- | --- |
| *Ec*NanX | *Escherichia coli* | P39352 |
| *Rc*NanX | *Rodentibacter caecimuris* | TGY51005.1 |
| *Hpa*NanX | *Haemophilus parainfluenzae* T3T1 | CBW14631.1 |
| *Hpi*NanX | *Haemophilus pittmaniae* | STO92481.1 |
| *At*NanX | *Anaerobiospirillum thomasii* | SPT69737.1 |
| *Se*NanX | *Salmonella enterica* | NP460104.1 |
| *Vp*NanX | *Vibrio ponticus* | OLQ84945.1 |
| *Ec*NanT | *Escherichia coli* | P41036 |
| *Yr*NanT | *Yokenella regensburgei* | QIU90626.1 |
| *Sb*NanT | *Shigella boydii* Sb227 | ABB67665.1 |
| *Se*NanT | *Salmonella enterica* | NP462248.1 |
| *Ck*NanT | *Citrobacter koseri* | WP_200076003.1 |
| *Ko*NanT | *Klebsiella oxytoca* | AUV90117.1 |
| *Ka*NanT | *Klebsiella aerogenes* KCTC 2190 | AEG95585.1 |
| *Yp*NanT | *Yersinia pestis* Angola | ABX87166.1 |
| *Asp.*NanT | *Actinopolyspora sp.* DSM 45965 | TQM88439.1\| |
| *Sa*NanT | *Streptomyces abyssalis* | OEU90734.1 |
| *Tsp.*NanT | *Tersicoccus* sp. Bi-70 | OMH36979.1 |
| *Mw*NanT | *Mycolicibacterium wolinskyi* | KWX25536.1 |
| *Mm*NanT | *Mycolicibacterium mageritense* DSM 44476 | CDO25105.1 |
| *Cac*NanT (J) | *Cutibacterium acnes* J139 | EFB87307.1 |
| *Cac*NanT (H) | *Cutibacterium acnes* HL110PA4 | EFT62140.1 |
| *Cg*NanT | *Corynebacterium gerontici* | AZA11325.1 |
| *Caq*NanT | *Corynebacterium aquilae* DSM 44791 | APT84973.1 |
| *Ph*NanT | *Pauljensenia hongkongensis* | AOS47187.1 |
| *So*NanT (F) | *Schaalia odontolytica* F0309 | EFF79992.1 |
| *So*NanT | *Schaalia odontolytica* | QGS10501.1 |

**
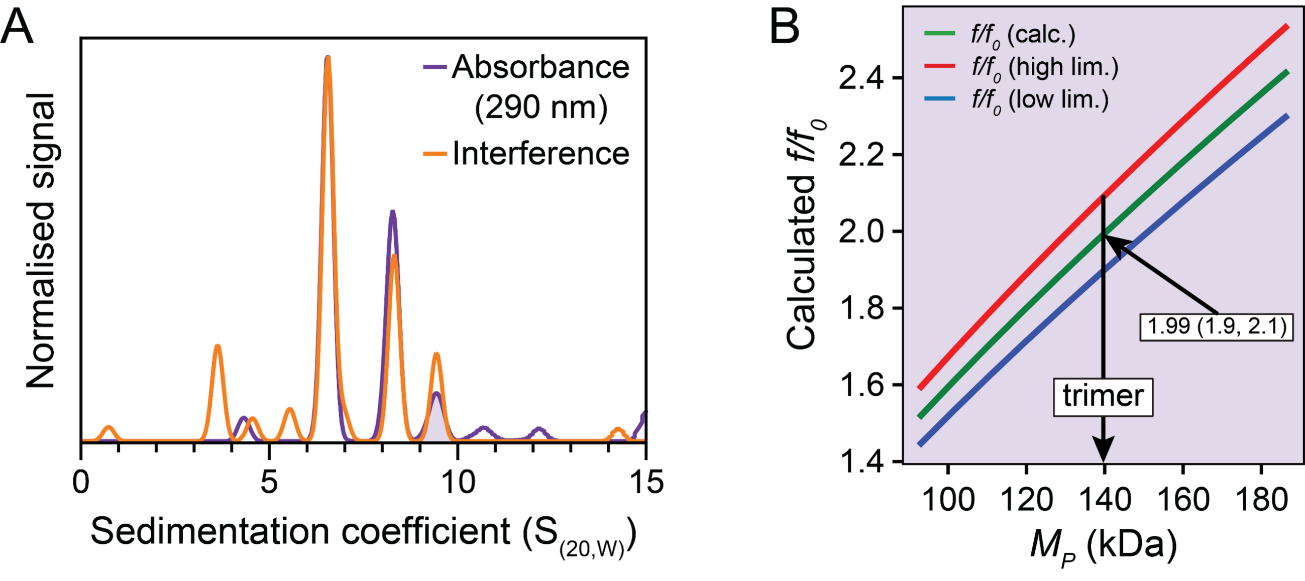
Figure S1 | Membrane protein calculations on the additional species observed in the analytical ultracentrifugation experiments with *Ec*NanX.** **A)** Sedimentation velocity analytical ultracentrifugation analysis using absorbance (290 nm, purple) and interference (orange) optics. **B)** The oligomeric state of the *Ec*NanX species at 9.4 S was calculated using the membrane protein calculation function in GUSSI [2], with the plot showing the calculated frictional ratio (*f*/*f*_0_) corresponding to the oligomeric state for the *Ec*NanX species at 9.4 S complexed with 414 molecules of lauryl maltose neopentyl glycol (LMNG). As the smaller species of *Ec*NanX corresponded to a monomer (6.6 S) and dimer (8.3 S), the next possible discrete *Ec*NanX oligomer was thought to be a trimer, however, the calculated *f*/*f*_0_ ratio of ~2, or complex with 414 molecules of LMNG is unlikely. This peak does not seem to correspond to a discrete oligomeric *Ec*NanX species based on the GUSSI calculations and its presence is likely due to the formation of aggregates over the course of the experiment. The analytical ultracentrifugation statistics and parameters used for the oligomeric state calculations are reported in **Tables S3** and **S4**, respectively.

**
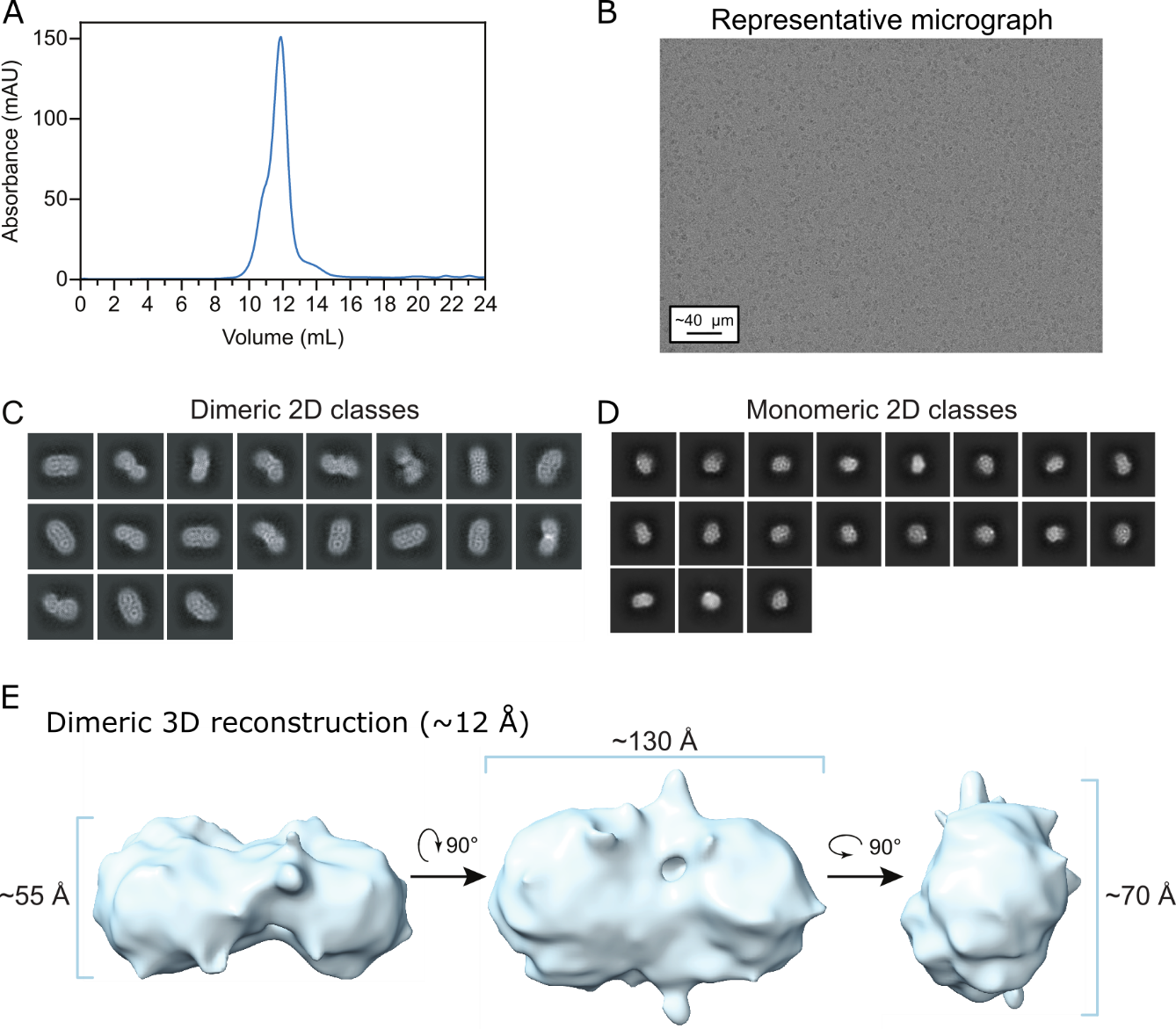
Figure S2 | Cryo-EM screening of *Ec*NanX. A)** Size-exclusion chromatography (SEC) elution profile of *Ec*NanX during the final SEC purification following amphipol exchange. **B)** Representative micrograph. **C)** A selection of 2D classes for the *Ec*NanX dimer. **D)** A selection of 2D classes for the *Ec*NanX monomer **E)** 3D reconstruction of the *Ec*NanX dimer contoured to 0.075 V, as calculated by ChimeraX [3]. The final resolution was ~12 Å and the particle was elongated consistent with an *Ec*NanX dimer with no distinguishing features outside the amphipol layer.

**
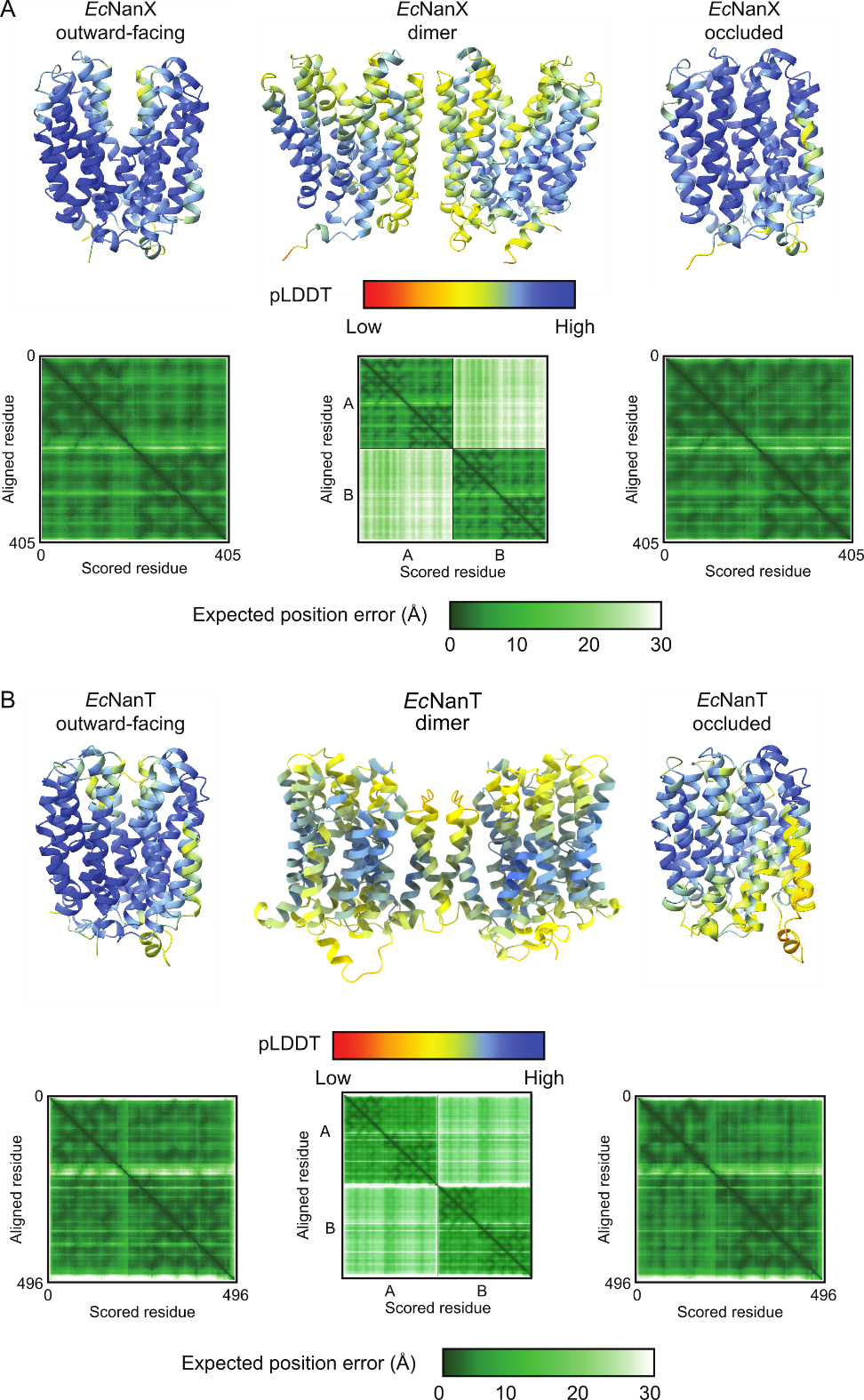
Figure S3 | Confidence metrics of the *Ec*NanX and *Ec*NanT models used in this study.** The predicted local distance difference test (pLDDT) and predicted aligned error (PAE) are shown for *Ec*NanX **(A)** and *Ec*NanT **(B)** AlphaFold2 models. In both cases, monomers display high intrachain confidence, while dimeric interfaces are low confidence. Dimeric models of NanX have interface predicted template modelling (ipTM) scores ranging 0.141–0.263, with the presented model (**A**) having an ipTM of 0.261. Dimeric models of NanT have ipTM scores ranging 0.147–0.469 with the presented model (**B**) having an ipTM of 0.469. Models are visualized with ChimeraX [3].

**
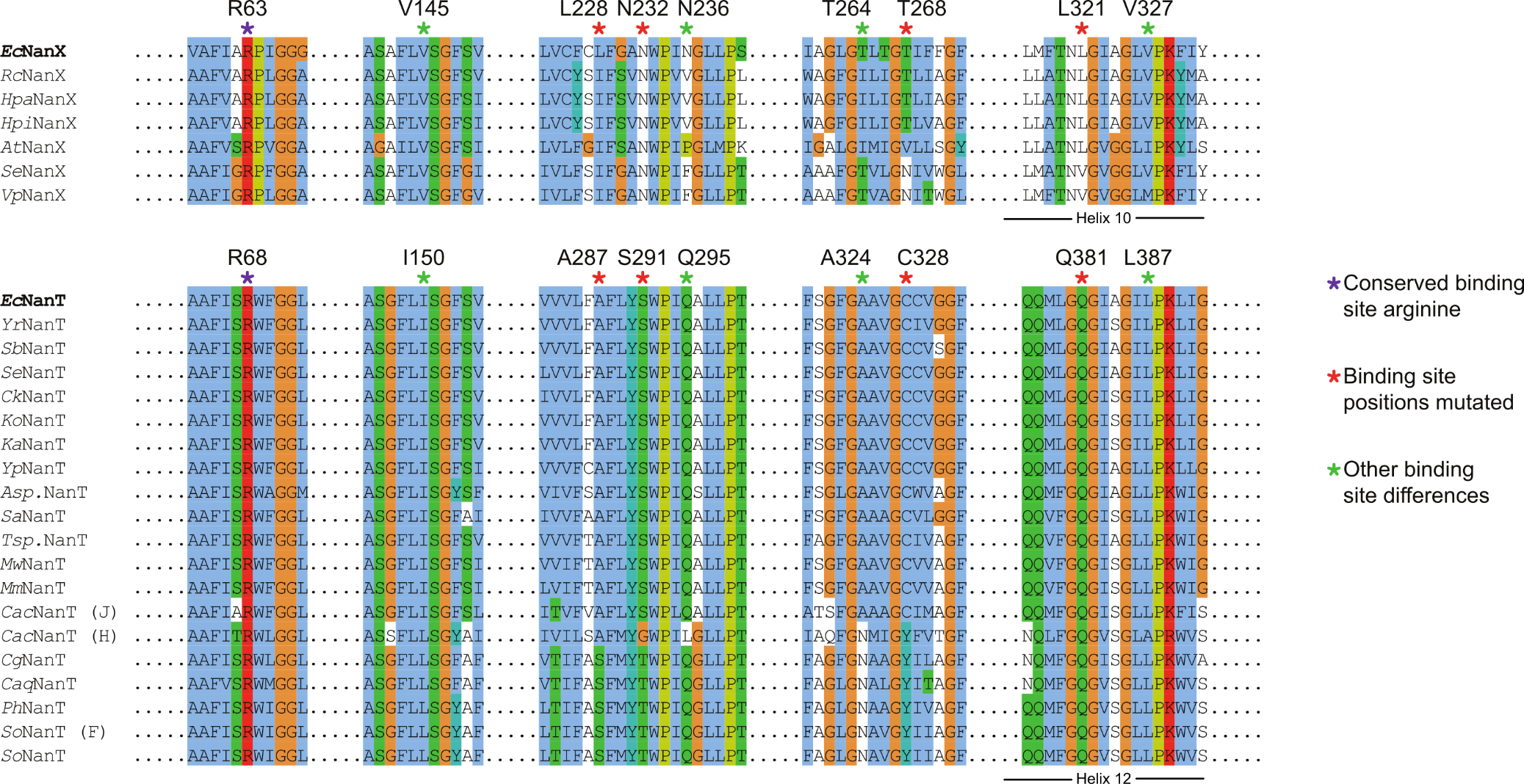
Figure S4 | Multiple sequence alignment of the NanX and NanT homologues identified by** **Severi et al. [4].** Residues that are different between *Ec*NanX and *Ec*NanT are indicated by stars, as well as the conserved arginine residue (purple star). The *Ec*NanX residues that were mutated are indicated with red stars and the other binding site residues that differed between *Ec*NanX and *Ec*NanT are indicated with green stars. The corresponding species and GenBank accession of each sequence are listed in **Table S5**. Helices 10 and 12 of NanX and NanT, respectively, are indicated as they are mentioned in reference to L321 and Q381. Qualifiers J, H, and F represent J139, HL110PA4, and F0309, respectively, and indicate bacterial strains. Alignment was generated with Jalview [5].

**
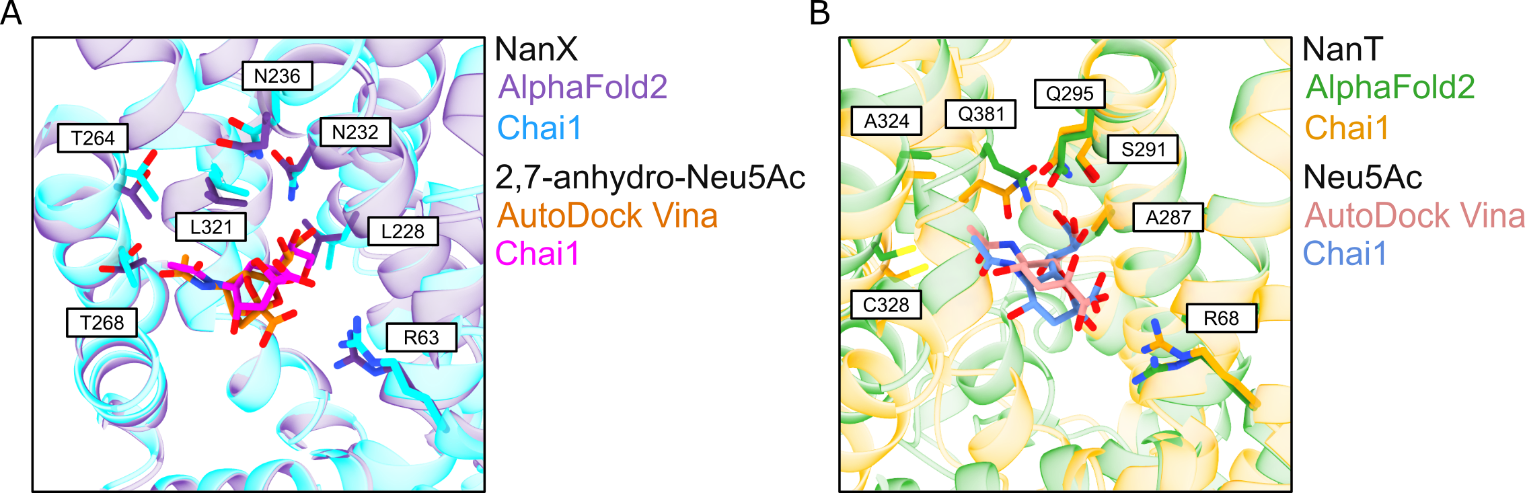
Figure S5 | Comparison of substrate binding using *in silico* methods. A)** Chai-1 [6] generated protein-ligand model (ipTM 0.65, pTM 0.87) of NanX and 2,7-Anhydro-*N*-acetylneuraminate (2,7-anhydro-Neu5Ac) places the substrate in a similar position and orientation as the AutoDock Vina [7] docked substrate into the occluded AlphaFold2 model. **B)** Similarly, for NanT-*N*-acetylneuraminate (Neu5Ac), the Chai-1 generated protein-ligand model (ipTM 0.57, pTM 0.82) largely matched both placement and orientation of Neu5Ac when docked into the AlphaFold2 model using AutoDock Vina. Models are visualized using ChimeraX [3].

**
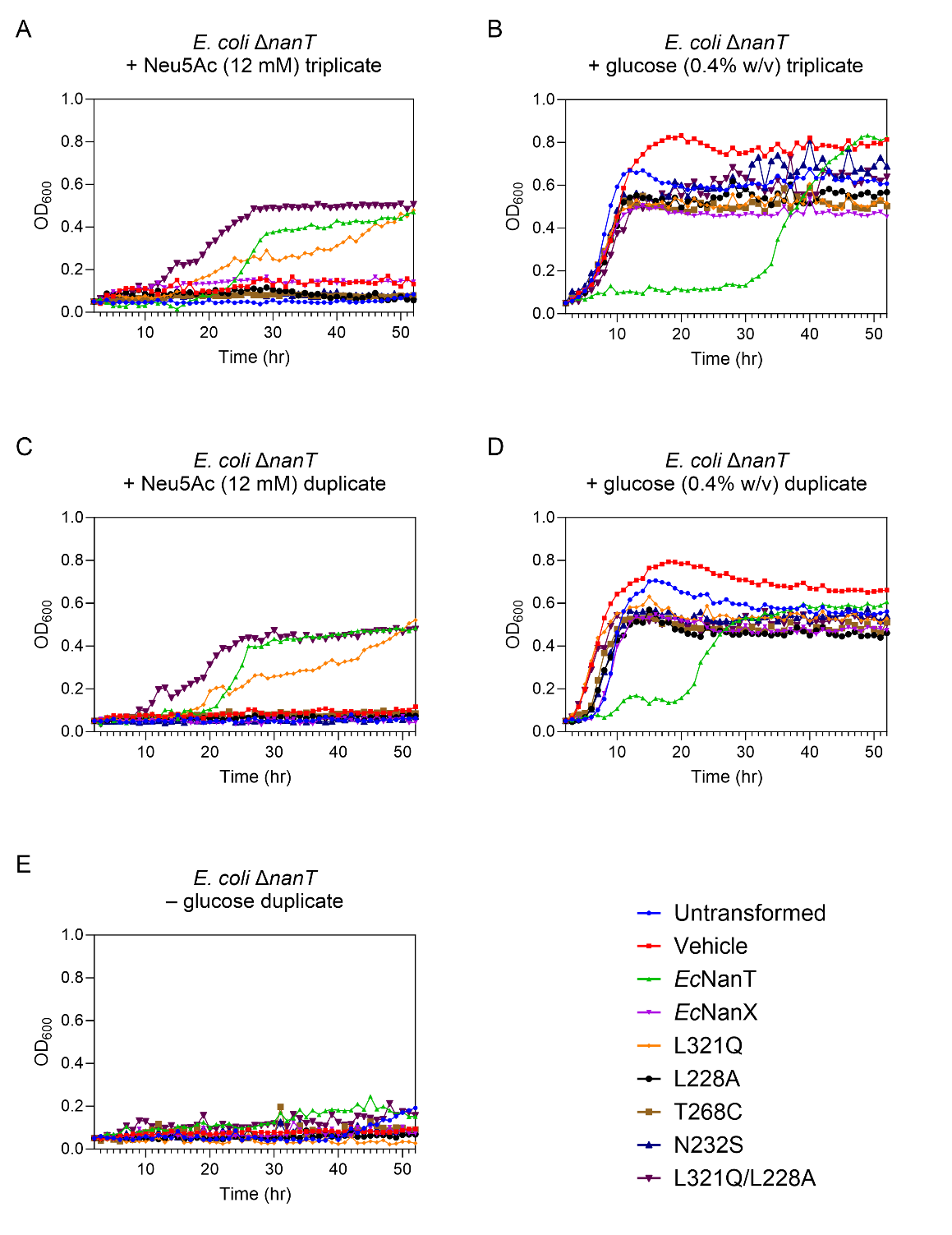
Figure S6 | Bacterial growth assay.** *E. coli* Δ*nanT* growth was tracked over 50 hours in M9 minimal medium with different carbon sources. OD_600_ starting baseline was adjusted to 0.05 and hourly measurements are presented. Individual experiments were conducted in technical triplicate (**A–B**) or duplicate (**C–E**). **A)** *E. coli* Δ*nanT* growth in 12.9 mM Neu5Ac; data are also presented in **Figure 6A**. **B)** *E. coli* Δ*nanT* growth in 0.4% w/v glucose; data are also presented in **Figure 6B**. **C)** Independent replicate in 12.9 mM Neu5Ac shows consistent results; data are also presented in **Figure 6A**. **D)** Independent replicate in 0.4 % w/v glucose further shows consistent results; data are also presented in **Figure 6B**. Note the delayed *Ec*NanT growth (green) in both + glucose panels (**B**, **D**). We surmise this may be due to toxicity in overexpression of *Ec*NanT. Pattern of growth delay is also apparent in Neu5Ac conditions (**A**, **C**). **E)** *E. coli* Δ*nanT* do not grow when there is no carbon source.

**Supplemental references**

1. Demeler, B. & Gorbet, G. E. (2016) Analytical ultracentrifugation data analysis with UltraScan-III in *Analytical Ultracentrifugation: Instrumentation, Software, and Applications* (Uchiyama, S., Arisaka, F., Stafford, W. F. & Laue, T., eds) pp. 119–143, Springer Japan, Tokyo.

2. Brautigam, C. A. (2015) Chapter five - calculations and publication-quality illustrations for analytical ultracentrifugation data in *Methods in Enzymol* (Cole, J. L., ed) pp. 109–133, Academic Press.

3. Meng, E. C., Goddard, T. D., Pettersen, E. F., Couch, G. S., Pearson, Z. J., Morris, J. H. & Ferrin, T. E. (2023) UCSF ChimeraX: tools for structure building and analysis, *Protein Sci.* **32**, e4792.

4. Severi, E., Rudden, M., Bell, A., Palmer, T., Juge, N. & Thomas, G. H. (2021) Multiple evolutionary origins reflect the importance of sialic acid transporters in the colonization potential of bacterial pathogens and commensals, *Microb Genom.* **7**.

5. Waterhouse, A. M., Procter, J. B., Martin, D. M. A., Clamp, M. & Barton, G. J. (2009) Jalview Version 2—a multiple sequence alignment editor and analysis workbench, *Bioinformatics.* **25**, 1189–1191.

6. Discovery, C. (2024) Chai-1: Decoding the molecular interactions of life, *bioRxiv*.

7. Eberhardt, J., Santos-Martins, D., Tillack, A. F. & Forli, S. (2021) AutoDock Vina 1.2.0: new docking methods, expanded force field, and python bindings, *J Chem Inf Model.* **61**, 3891–3898.
